# The Effect of Eighteen-Month Metformin Treatment in Obese Adolescents: Comparison of Results Obtained in Daily Practice with Results from a Clinical Trial

**DOI:** 10.1155/2016/7852648

**Published:** 2016-12-22

**Authors:** Marloes P. van der Aa, Vera Hoving, Ewoudt M. W. van de Garde, Antonius de Boer, Catherijne A. J. Knibbe, Marja M. J. van der Vorst

**Affiliations:** ^1^Department of Pediatrics, St. Antonius Hospital, Nieuwegein/Utrecht, P.O. Box 2500, 3430 EM Nieuwegein, Netherlands; ^2^Department of Clinical Pharmacy, St. Antonius Hospital, Nieuwegein/Utrecht, P.O. Box 2500, 3430 EM Nieuwegein, Netherlands; ^3^Division of Pharmacoepidemiology and Clinical Pharmacology, Utrecht Institute for Pharmaceutical Sciences (UIPS), Utrecht University, P.O. Box 80082, 3508 TB Utrecht, Netherlands

## Abstract

*Background*. In a recent randomized controlled trial (RCT) in obese adolescents, 18 month-treatment with metformin versus placebo was reported to lead to stabilisation of the BMI. This study aimed to compare the effect of metformin on BMI in obese adolescents in daily practice versus results obtained in an RCT.* Methods*. Obese adolescents treated off label with metformin in daily practice in an outpatient clinic with a follow-up of ≥18 months were identified. Anthropometric and biochemical data were collected at baseline and at 18 months. Patients treated with metformin for 18 months in an RCT were used for comparison. BMI was compared between the two groups.* Results*. Nineteen patients (median age 14.3 (interquartile range 11.7–15.7) years, BMI 31.3 (28.8–33.8) kg/m^2^) treated in daily practice were compared to 23 patients receiving metformin in the RCT (age 13.6 (12.6–15.3) years, BMI 29.8 (28.1–34.5) kg/m^2^). BMI change after 18 months was −0.36 (−2.10–1.58) versus +0.22 (−2.87–1.27) kg/m^2^ for the two groups, respectively. In the multivariable model, BMI change was not statistically significantly different between the two groups (*p* = 0.61).* Conclusion*. Treatment with metformin in obese adolescents in daily practice resulted in a comparable change in BMI as observed in an RCT. This trial is registered with ClinicalTrials.gov number: NCT01487993.

## 1. Introduction

Childhood obesity is rising, as well as attention for obesity treatments. Cornerstone in the treatment of obesity is lifestyle intervention that has proven to lead to a decrease in body mass index (BMI) after 6–12 months [1]. Longer-term effects have been described to be marginal additive [2]. To potentially improve the effects of lifestyle interventions, additional pharmacological interventions have been suggested and studied [3–5].

Metformin is one of these pharmacological agents used in adolescents with obesity. Metformin is registered for the treatment of type 2 diabetes in children aged 10 years and older. It is frequently used off label for the treatment of children with obesity. In a systematic review and meta-analysis, a reduction in BMI of −1.38 (95% CI −1.93–−0.82) kg/m^2^ was reported for metformin after 6 months of treatment [6]. However, the effect after >12 months of treatment was not significantly different compared to placebo [6]. As such, it seems that the maximum effect of metformin is achieved after 6–9 months of treatment, since in studies of 48 weeks and 18 months smaller effects for change in BMI were reported [7, 8]. In our RCT of 18 months, BMI decreased during the first 9 months of metformin treatment, comparable to the results in the study of 48 weeks. After 18 months, the BMI returned back to baseline in the metformin group with an increase compared to baseline in the placebo group.

Generally, it is believed that patients who participate in a trial are more likely to change their behaviour, because they are frequently monitored. This phenomenon is also known as the Hawthorne effect [9, 10]. The Hawthorne effect is considered as one of the explanations for improvements in health in clinical trials. As a result, the effects observed in clinical trials might be larger than the results that can be obtained in daily clinical practice. Such a difference in effect can also be referred to as the efficacy-effectiveness gap. The aim of the present study is to compare the effects of metformin treatment in addition to lifestyle intervention on change in BMI between obese adolescents treated with metformin in daily clinical care and obese adolescents treated with metformin as participants to a corresponding randomized placebo controlled trial (RCT). In addition, the effects on glucose metabolism between the two groups are compared.

## 2. Materials and Methods

### 2.1. Patients

In this study, two groups of patients were compared, that is, the daily clinical practice group and the RCT group. The daily clinical practice group consisted of patients who were treated off label with metformin in the pediatric obesity outpatient clinic of the St. Antonius Hospital, Nieuwegein/Utrecht, between January 1 2007 and July 31 2015. The data of these patients were collected retrospectively. The patients in the daily clinical practice group were included if they were aged 10–16 years at start of metformin therapy, were obese (defined as BMI standard deviations score (BMI-SDS) > 2.3), and had a follow-up time of at least 18 months. According to the intention to treat principle, patients should have started metformin, but treatment with metformin for the complete 18-month follow-up was not required. Metformin doses in the daily practice groups were 1000 mg twice daily or in case of gastrointestinal side-effects 500 mg twice daily (*n* = 4) or 1000 mg once a day (*n* = 1). Patients were excluded if they had type 2 diabetes mellitus. As standard of care, to all patients in the obesity outpatient clinic a multidisciplinary lifestyle intervention programme is offered. Ethical approval of the study protocol (Protocol number Z-11.27) was obtained from the local Medical Ethical Committee of the St. Antonius Hospital. As only routinely collected information was used and analysed anonymously, the need for written informed consent of the children and their parents was waived.

The other group, that is, the RCT group, consisted of the patients of the metformin arm of a RCT on metformin versus placebo in obese children [8, 11]. The inclusion criteria for the patients in the RCT were age 10–16 years, obesity (defined as BMI-SDS > 2.3), insulin resistance (defined as HOMA-IR ≥ 3.4), and being of Caucasian origin. The Medical Ethical Committee of the St. Antonius Hospital, Nieuwegein/Utrecht, the Netherlands, approved the RCT study protocol and written informed consent was obtained from participants (if applicable) and parents.

### 2.2. Data Collection

**Figure 1:**
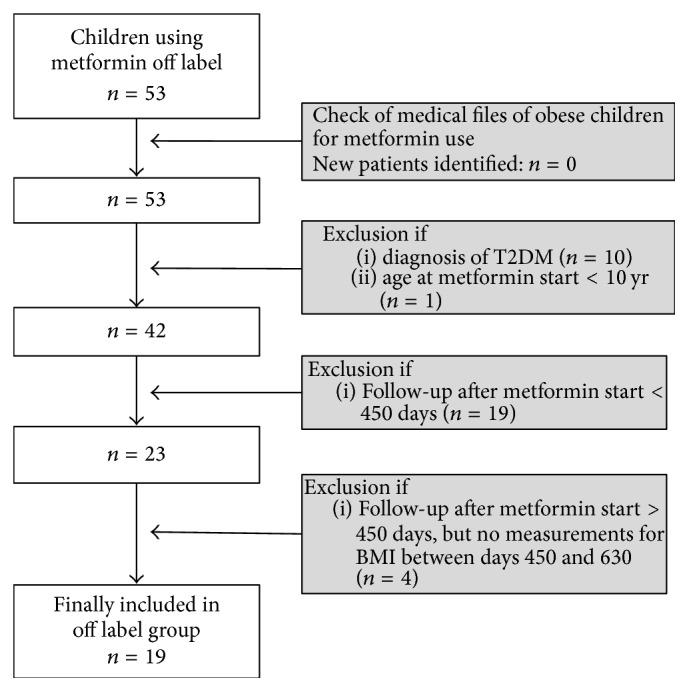
Flowchart of included patients in the daily clinical practice group.

For the daily clinical practice group, the outpatient pediatrician identified the patients in the outpatient clinic after which a double check was performed by identifying all patients who visited the pediatric obesity outpatient clinic between 2006 and 2014 using the “diagnose behandel combinatie” (diagnosis treatment combination-code (DBC-code)) “adiposity.” The medical files of these obese children were screened for treatment with metformin. No additional patients were identified (Figure 1).

From the included patients, data were extracted from the electronic patient files. Baseline (*t* = 0) was defined as date of start of metformin therapy. Collected baseline data were age, gender, height (cm), weight (kg), fasted plasma glucose (FPG) in mmol/l, fasted plasma insulin (FPI) in mU/l, and HbA1c in mmol/mol. BMI was calculated from height and weight (BMI = weight (kg)/(height (m))^2^). BMI-SDS was calculated by the “TNO Groeicalculator voor professionals” (https://groeiweb.pgdata.nl/calculator.asp), which is a web application developed by the Dutch Organisation for Applied Scientific Research (TNO) calculating age and gender adjusted height and BMI standard deviation scores. Impaired fasted glucose was defined as FPG ≥ 5.6 mmol/l and hyperinsulinemia as FPI > 15 *μ*U/ml. For insulin resistance, the Homeostasis Model Assessment for Insulin Resistance (HOMA-IR) was calculated: (FPG (mmol/l) *∗* FPI (mu/l))/22.5 [12]; insulin resistance was defined as HOMA-IR ≥ 3.4.

Since subjects did not regularly visit the obesity outpatient clinic, windows were created to define times of visit. The windows were *t* = 6 months (day 180 (range 120–240)), *t* = 12 months (day 360 (range 300–420)), and *t* = 18 months (day 540 (range 450–630)). Data extracted from follow-up visits were date of visit, height (cm), weight (kg), FPG in mmol/l, FPI in mU/l, and HbA1c in mmol/mol.

Patients participating in the RCT visited the outpatient clinic every three months. During these visits height (cm) and weight (kg) were measured and with these data BMI and BMI-SDS were calculated. Vena punctures were performed every visit to measure FPG and FPI (every 3 months) and HbA1c (every 6 months). Patients received metformin 1000 mg twice daily; physical training by a physical therapist was offered twice weekly. A detailed description of this group is described elsewhere [11].

### 2.3. Statistical Analysis

Since most parameters were not normally distributed in the RCT group, all data were reported as median (interquartile range). Baseline characteristics of continuous data were compared using the Mann-Whitney *U* test. For dichotomous data the chi squared test was used. The change in BMI (ΔBMI) and BMI-SDS (ΔBMI-SDS) between baseline and *t* = 18 months was compared between the two groups (off label treatment versus RCT-treatment) using the Mann-Whitney *U* test. Subsequently, a multivariable linear regression analysis was conducted to assess the effect of trial participation (yes/no) adjusted for potential confounding factors. The latter model was constructed in triple, with ΔBMI, ΔBMI-SDS, and ΔFPG as outcomes of interest. All variables that differed at baseline between the two groups (*p* < 0.10) were considered as potential confounders and added stepwise to the model. Variables were retained in the final regression model if the coefficient of trial participation changed >10%. In case of missing data regarding the outcome of interest, the case was excluded from that analysis. Results were considered statistically significant if *p* values are <0.05. All analyses were performed using SPSS version 22.0 (IBM Corp., Armonk, NY, USA).

## 3. Results

### 3.1. Patients

In the daily clinical practice group, 19 patients were identified who were treated with metformin for 18 months (Figure 1). For the RCT group, the data of 23 patients were eligible for analysis. In Table 1 baseline characteristics of both groups are displayed. In the daily clinical practice group, more boys (57.9%) than girls (42.1%) were included, whereas the participants in the RCT were more girls (73.9%) than boys (26.1%). The participants in the daily clinical practice group were of multiethnic origin, with 11/19 (58%) being Caucasian. Other ethnicities were Asian (*n* = 2), African (*n* = 2), North African (*n* = 2), and Hindustani (*n* = 2). In the RCT all participants were of Caucasian origin as a result of the inclusion criteria. Both groups were equal in age, height, weight, and BMI at baseline. Morbid obesity (defined as BMI-SDS > 3.0) was more prevalent in the daily clinical practice group; that is, 15/19 (78.9%) were morbidly obese, versus 13/23 (56.5%) in the RCT group, which was not significant (*p* = 0.13). Baseline differences were observed for FPG, FPI, and HOMA-IR, with significantly higher prevalence of impaired fasted glucose (FPG ≥ 5.6 mmol/l), hyperinsulinemia (FPI > 15 *μ*U/ml), and insulin resistance (HOMA-IR ≥ 3.4) in the daily clinical practice group (Table 1).

### 3.2. Change in BMI over 18 Months

**Figure 2:**
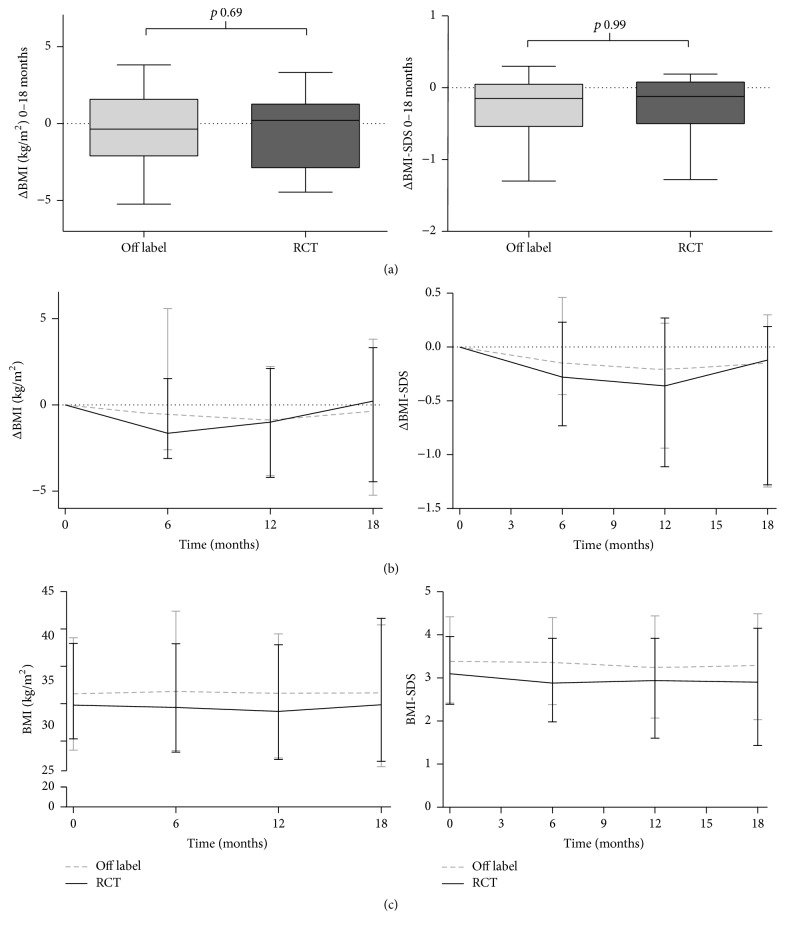
Change in BMI (left column) and BMI-SDS (right column) over 18 months of treatment. (a) Change between baseline and *t* = 18 months. (b) Median ΔBMI (SDS) over time; (c) Median BMI ( SDS) over time. Graphs (b) and (c) represent median (min-max).

In Table 2, the results after 18 months of treatment are presented. Median ΔBMI over 18 months in the daily clinical practice group was −0.36 (−2.10–1.58) kg/m^2^, versus +0.22 (−2.87–1.27) kg/m^2^ in the RCT group, which is not a statistically significant difference (*p* = 0.69) (Figure 2). The corresponding changes in BMI-SDS were −0.15 (−0.54–−0.05) and −0.12 (−0.50–0.08) for the off label and RCT group, respectively (*p* = 0.99) (Figure 2). In the multivariable linear regression analyses, study participation was not associated with ΔBMI nor ΔBMI-SDS. Variables that influenced the coefficient for study participation with more than 10% were gender in the ΔBMI model and gender, height, and insulin resistance for BMI-SDS, with final regression coefficients of −0.40 (−1.93–1.13) (*p* = 0.61) and −0.02 (−0.31–0.28) (*p* = 0.90), respectively.

### 3.3. Change in Glucose Metabolism over 18 Months

**Figure 3:**
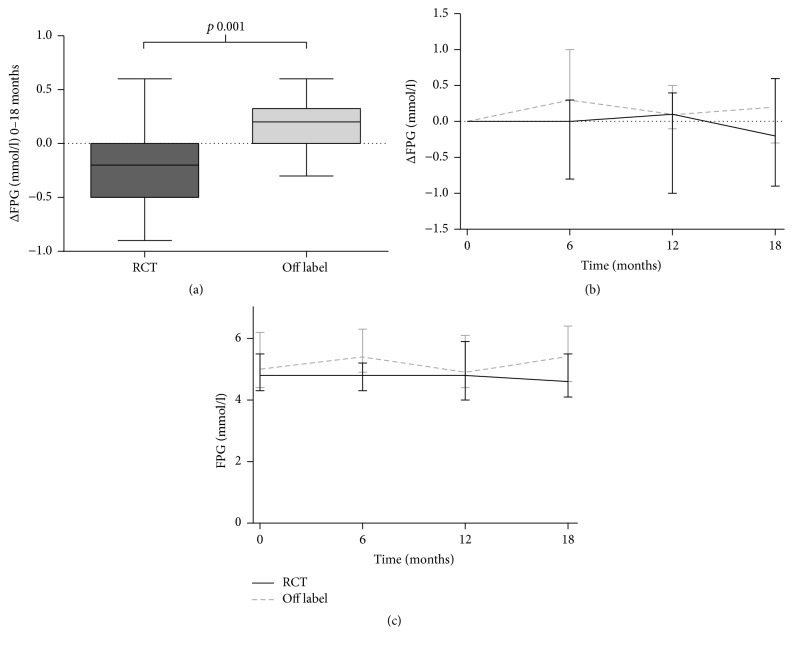
Change in fasted plasma glucose. (a) Change between baseline and *t* = 18 months. (b) Median ΔFPG over time. (c) Median FPG over time. Graphs (b) and (c) represent median (min-max).

At baseline, the daily clinical practice group had significant higher levels of FPG (Table 1) while impaired fasted glucose was present in 4/19 patients, versus 0/23 patients in the off label versus the RCT group. Univariate analysis of the ΔFPG showed a significant difference between both groups, with an increase of +0.2 (0.0–0.3) mmol/l in the daily clinical practice group and a decrease of −0.2 (−0.5–0.0) mmol/l in the RCT group (*p* = 0.001) (Figure 3). This remained significant in a multivariate analysis model containing IR at baseline (*p* < 0.001). For ΔFPI, ΔHOMA-IR, and ΔHbA1c no significant difference between the groups was observed (Table 2).

## 4. Discussion

In this observational study, we compared treatment results of metformin in obese adolescents treated in daily clinical practice in an outpatient pediatric obesity clinic with results of a RCT in obese adolescents. We observed that metformin treatment in obese adolescents in daily clinical practice was associated with change in BMI similar to the change during metformin treatment in obese adolescents in a RCT.

The RCT was the first study reporting on the effects of metformin versus placebo during 18-month treatment, showing a ΔBMI of +0.22 (−2.87–1.27) kg/m^2^ in the metformin group. This small increase in ΔBMI at 18 months was initially preceded by a substantial decrease in ΔBMI in the metformin arm at 9 months, while in the placebo arm of this RCT ΔBMI from baseline to 18 months was found to increase significantly compared to the metformin arm (i.e., ΔBMI +1.17 (−0.26–2.37) kg/m^2^ (*p* = 0.015)) [8]. In the current study, the course of BMI and BMI-SDS over time in the daily clinical practice group also showed an initial decrease in BMI and BMI-SDS in accordance with the RCT group. After 6–12 months, the median BMI and BMI-SDS started to increase again (Figures 2 and 3), which could be an indication that the effect of metformin fades out after a certain period of treatment. This is in line with the findings in the meta-analysis of McDonagh et al., where the effect after >6 months was −0.79 (95% CI −1.63–0.06) kg/m^2^ compared to 6 months of treatment −1.38 (95% CI −1.93–−0.82) kg/m^2^ [6]. Although the effect of metformin might fade out over time, it remains unclear whether prolonged use (>18 months) of metformin is not effective any more (i.e., children treated with metformin return to their previous BMI-percentile), or whether it will result in persisting lower BMI-values compared to placebo. For the 18-month treatment, the current study showed that the change in BMI upon metformin in daily clinical practice was similar to results as obtained in a RCT after treatment of 18 months [8].

In contrast to the ΔBMI, the ΔFPG was different between both groups after 18 months, with an increase in FPG in the daily clinical practice group. Next to ΔFPG, baseline FPG, FPI, and HOMA-IR were significantly higher in this group compared to the RCT group. In particular the difference in HOMA-IR is remarkable, since inclusion in the RCT required a HOMA-IR ≥ 3.4, while no criteria for HOMA-IR were used for the daily clinical practice group. Selection bias for treatment with metformin in daily clinical practice is a possible explanation. The clinician might tend to reserve off label treatment with metformin for children with seriously increased levels of FPG, FPI, or HOMA-IR. Another explanation could be the difference in ethnicity between both groups. The participants of the RCT were all Caucasian, whereas the daily clinical practice group was multiethnic. Since some ethnicities are at higher risk for T2DM than others, for example, African Americans, Asians, and South Indians [13], this might result in higher prevalence rates of T2DM precursors in these groups. In our daily clinical practice population 5 children had impaired fasted glucose at baseline, of which 2 were of Hindustan origin and 2 of North African (Moroccan) origin. Regarding the influence of ethnicity on the effect of metformin, a study by Williams et al. found a better glycemic response in African Americans compared to European Americans [14]. Nagi and Yudkin found no difference in effect of metformin in Caucasian and Asian subgroups [15]. Based on these studies the influence of ethnicity on the glycemic response to metformin can neither be confirmed nor be ruled out. Therefore, the difference in ΔFPG in our study remains not well explained, and since in the RCT group all participants are Caucasian further analysis of our data was not possible.

A limitation of our study is the retrospective data collection in the daily clinical practice group. For FPG, FPI, and HOMA-IR this resulted in 26% and for HbA1c 68% missing data after 18 months of treatment, despite the use of time windows for the visits after 6, 12, and 18 months, ultimately reducing our patient number from 19 to 14 patients for the analysis of FPG, FPI, and HOMA-IR and 6 patients for HbA1c. As a benefit of the retrospective data collection in the outpatient clinic, patients were not aware of being studied.

Another limitation is the incomplete information on lifestyle intervention of the daily clinical practice group. In daily clinical practice a lifestyle intervention programme is offered as standard care to all patients but is not clear whether all patients attended these programmes and whether all programmes were comparable. Some patients in daily clinical practice received dietary advice by a dietician, whereas others received limited dietary advice by the paediatrician. To all participants of the RCT a lifestyle programme consisting of dietary advice and physical training twice weekly was offered.

## 5. Conclusions

In this study BMI remained stable over 18 months in adolescents in daily practice, which is comparable to the results obtained under the strict circumstances of a RCT. It is reassuring that metformin added to lifestyle interventions in daily practice is associated with a similar change in BMI as observed during metformin use in experimental conditions.

## Figures and Tables

**Table 1:** Baseline characteristics of patients treated with off label metformin and treated with metformin in a randomized clinical trial.

	Daily clinical practice group (*n* = 19)	RCT group (*n* = 23)	*p* value chi^2^	*p* value Mann-Whitney
Gender				
(i) Boys	11 (57.9)	6 (26.1)	**0.037**	
(ii) Girls	8 (42.1)	17 (73.9)		
Age (years)	14.3 (11.7–15.7)	13.6 (12.6–15.3)		0.99
Ethnicity				
(i) Caucasian	11 (57.9)	23 (100)	NA	
(ii) Other	8 (42.1)	0 (0)		
Height (cm)	168.3 (161.5–177.2)	162.9 (159.9–168.0)		0.08
Weight (kg)	92.5 (75.2–104.0)	82.2 (75.4–92.7)		0.16
BMI (kg/m^2^)	31.3 (28.8–33.8)	29.8 (28.1–34.5)		0.66
BMI-SDS	3.23 (3.05–3.64)	3.10 (2.72–3.52)		0.37
BMI-SDS ≥ 3.0	15 (78.9)	13 (56.5)	0.13	
Tanner stage				
(i) Prepubertal (TS1)	3 (15.8)	3 (13.0)	0.42	
(ii) Pubertal (TS2–4)	5 (26.4)	17 (74.0)		
(iii) Postpubertal (TS5)	1 (5.6)	3 (13.0)		
(iv) Unknown	10 (52.6)	0 (0)		
FPG (mmol/l)	5.0 (4.8–5.6)	4.8 (4.7–5.0)		**0.015**
FPG ≥ 5.6 mmol/l	5 (26.3)	0 (0)	NA	
FPI (*μ*U/ml)	31.0 (22.0–41.9)	18.0 (11.0–27.0)		**0.005**
FPI > 15 *μ*U/ml	17 (89.5)	14 (60.9)	**0.036**	
HOMA-IR	7.74 (4.48–8.96)	4.00 (2.30–6.36)		**0.003**
HOMA-IR ≥ 3.4	17 (89.5)	10 (43.5)	**0.019**	
HbA1c (mmol/mol)	36 (32–39)^#^	33 (31–34)		0.052

Data are presented as median (interquartile range) or *n*(%). ^#^*n* = 14.

RCT: randomized clinical trial; BMI ( SDS): body mass index (standard deviation score); FPG: fasted plasma glucose; FPI: fasted plasma insulin; HOMA-IR: homeostasis model assessment for insulin resistance; NA: not applicable.

**Table 2:** Results after 18 months of treatment.

	Daily clinical practice group (*n* = 19)			RCT group (*n* = 23)			Delta
	*t* = 0	*t* = 18	Δ*t* = 18 − *t* = 0	*t* = 0	*t* = 18	Δ*t* = 18 − *t* = 0	*p* value
BMI (kg/m^2^)	31.0 (27.9–32.8)	30.5 (26.0–32.4)	−0.36 (−2.10–1.58)	29.8 (28.1–34.5)	29.9 (26.3–33.6)	0.22 (−2.87–1.27)	0.686
BMI-SDS	3.23 (3.05–3.64)	3.00 (2.43–3.37)	−0.15 (−0.54–0.05)	3.10 (2.72–3.52)	2.90 (2.34–3.39)	−0.12 (−0.50–0.08)	0.990
FPG (mmol/l)	5.0 (4.8–5.6)	5.4 (5.0–5.7)^a^	0.2 (0.0–0.3)^a^	4.8 (4.7–5.0)	4.6 (4.4–4.8)	−0.2 (−0.5–0.0)	**0.001**
FPI (*μ*U/ml)	31.0 (21.0–41.9)	19.6 (11.0–34.0)^a^	−5.0 (−20.5–6.3)^a^	18.0 (11.0–27.0)	15.0 (10.0–20.0)	−3.0 (−13.0–6.0)	0.661
HOMA-IR	7.16 (4.31–8.96)	4.29 (2.52–9.43)^a^	−1.03 (−4.48–1.88)^a^	4.00 (2.30–6.36)	3.00 (2.00–4.29)	−1.00 (−3.17–1.47)	0.802
HbA1c	34 (32–39)^a^	34 (33–36)^a^	−1.0 (−3.5–3.5)^b^	33 (31–34)	34 (31–34)^c^	1.0 (−1.0–2.3)^c^	0.480

Data are presented as median (interquartile range). ^a^*n* = 14; ^b^*n* = 6; ^c^*n* = 22.

BMI (SDS): body mass index (standard deviation score); FPG: fasted plasma glucose; FPI: fasted plasma insulin; HOMA-IR: homeostasis model assessment for insulin resistance.
